# Administration of Essential Phospholipids Prevents *Drosophila Melanogaster* Oocytes from Responding to Change in Gravity

**DOI:** 10.3390/cells13181593

**Published:** 2024-09-22

**Authors:** Ksenia K. Gogichaeva, Irina V. Ogneva

**Affiliations:** 1 Cell Biophysics Laboratory, State Scientific Center of the Russian Federation Institute of Biomedical Problems of the Russian Academy of Sciences, 76 a, Khoroshevskoyoe Shosse, 123007 Moscow, Russia; xeniagogichaeva@gmail.com; 2 Medical and Biological Physics Department, I.M. Sechenov First Moscow State Medical University, 8-2 Trubetskaya Street, 119991 Moscow, Russia

**Keywords:** mechanoreception, weightlessness, hypergravity, oocyte, cholesterol, *Drosophila melanogaster*

## Abstract

The aim of this study was to prevent initial changes in *Drosophila melanogaster* oocytes under simulated weightlessness and hypergravity at the 2 g level. Phospholipids with polyunsaturated fatty acids in the tail groups (essential phospholipids) at a concentration of 500 mg/kg of nutrient medium were used as a protective agent. Cell stiffness was determined using atomic force microscopy, the change in the oocytes’ area was assessed as a mark of deformation, and the contents of cholesterol and neutral lipids were determined using fluorescence microscopy. The results indicate that the administration of essential phospholipids leads to a decrease in the cholesterol content in the oocytes’ membranes by 13% (*p* < 0.05). The stiffness of oocytes from flies that received essential phospholipids was 14% higher (*p* < 0.05) and did not change during 6 h of simulated weightlessness or hypergravity, and neither did the area, which indicates their resistance to deformation. Moreover, the exposure to simulated weightlessness and hypergravity of oocytes from flies that received a standard nutrient medium led to a more intense loss of cholesterol from cell membranes after 30 min by 13% and 18% (*p* < 0.05), respectively, compared to the control, but essential phospholipids prevented this effect.

## 1. Introduction

The progress of humanity in the future may be associated with the exploration of space beyond Earth’s orbit. The latter will inevitably be accompanied by alternating effects of weightlessness, overload, and other examples of gravity. Today, there is no doubt that space flight leads to the development of a complex of negative changes in key systems of the body, in particular in the cardiovascular [1,2,3,4], nervous [5,6], and musculoskeletal [7,8,9,10,11] systems, for the relief of which a regimen of physical training and a special diet are most often used [12,13].

However, numerous studies of various organisms and tissue types under conditions of weightlessness and overload indicate that the vast majority of changes that occur are based on changes at the cellular level, in particular in skeletal muscle fibers and cardiomyocytes [14,15,16,17,18], osteocytes [19], and neurons [20,21]. Accordingly, it can be assumed that the search for fundamentally new ways to protect the human body during deep space exploration should begin with a single cell.

In a number of experiments, primarily in suborbital and space flights, it was shown that a change in gravity almost instantly leads to various structural and functional changes in cells [22,23], and the main participant in these processes is the cytoskeleton, changes in which are also well documented [24,25,26,27,28,29,30]. To study single-cell gravireception, we selected single oocytes from the fruit fly *Drosophila melanogaster*. These are convenient models because they can be displayed on wet agar plates and, thus, avoid artifacts due to fluid shear stresses. Our previous results show that simulated weightlessness and hypergravity at the first stage of mechanotransduction lead to different cell deformations, which, in turn, leads to the migration of various cytoskeletal proteins from the submembrane cytoskeleton, and, as a result, cell stiffness decreases [31,32,33].

Therefore, according to the laws of mechanics, we can expect that the rise in cell stiffness will prevent or reduce the response of a single cell to a change in the gravity field and acting on it. Indeed, the condensation of the cortical cytoskeleton with calyculin A prevented changes in oocyte stiffness in simulated weightlessness and hypergravity [33]. On the other hand, it has been shown that the extraction of cholesterol from the cell membrane of fibroblasts (but not cancer cell lines) with methyl-beta-cyclodextrin increases the F-actin content in the cortical cytoskeleton and makes the membrane–cortical cytoskeleton structure stiffer [34,35]. However, the use of these drugs in vivo is not possible.

At the same time, changes in the composition of the cell membrane can be achieved by using phospholipids with polyunsaturated fatty acids in the tail groups, called essential phospholipids [36]. Lipids with such tail groups occupy a larger volume and the efficiency of formation of cholesterol rafts, as well as their maintenance, decreases. And we could expect an increase in cell stiffness due to strengthening of the cortical cytoskeleton. In female mice, the use of such phospholipids led to a decrease in the cholesterol content and prevented changes in the profile of cytoskeletal proteins in oocytes when these cells were exposed to simulated weightlessness for 6 h [37].

In this work, we tested the hypothesis that increasing the cell stiffness would prevent cell deformation in an altered gravitational field and assessed the possibility of using essential phospholipids for this purpose. As before, oocytes of the fruit fly *Drosophila melanogaster* were used, but of two types: some were collected from flies that received a standard nutrient medium, and others were collected from flies whose previous three generations were cultured on a nutrient medium supplemented with essential phospholipids.

## 2. Materials and Methods

### 2.1. Experimental Design

The study used mature oocytes (stage S14) of two types of virgin fruit fly *Drosophila melanogaster* line Canton S: one was cultured on a standard nutrient medium; the other was cultured for at least three previous generations on a standard nutrient medium with the addition of phospholipids containing polyunsaturated fatty acids in the tail groups, called essential phospholipids, as commercially available EssentsialeR Forte N (A. Nattermann and Cie. GmbH, Germany) (designated as “+E”) at a concentration of 500 mg/kg of nutrient medium, according with our previous experiments [37].

As previously described [33], virgin 5-day-old females of both types were placed into the cages with agar plates, and oocytes were collected and dechorionized using sodium hypochlorite as a 50% bleach [38]. Next, the oocytes were placed on agar plates in a humidified atmosphere, which were randomly divided into control (C and C + E) and experimental plates that would be exposed to microgravity (sµg and sµg + E) or hypergravity (hg and hg + E). To simulate micro- and hypergravity, the plates were placed on a Gravite platform (Gravite^®^, GC-US-RCE010001, Space Bio-Laboratories Co., Ltd., Hiroshima, Japan) for 30, 90, 210, and 360 min. We used dechorionized oocytes and limited the exposure time to 360 min, which is the standard incubation time in in vitro fertilization protocols. The environmental conditions for the experimental and control plates with oocytes were the same.

There were at least 100 oocytes in each study group, and for each experimental result, there were at least three biological replicas. All the experimental procedures were approved by the Commission on Biomedical Ethics of the SSC RF Institute of Biomedical Problems (Minutes No. 624, dated 20 October 2022).

### 2.2. Stiffness Measurements Using Atomic Force Microscopy

The measurement of the oocytes’ stiffness was carried out as before [30,33] using the atomic force microscope NTEGRA NEXT II (NT-MDT, Moscow, Russia) with a soft silicon cantilever (CS17, NT-MDT, Moscow, Russia). The resonance curve was recorded, and the resonant frequency of the cantilever’s own oscillations and, accordingly, its own stiffness were determined, which amounted to 87.8 pN/nm. After exposure, the oocytes of each study group were placed on a rigid substrate; in contact mode, a calibration curve was obtained outside the oocytes, and then force curves were recorded for the oocytes. The indentation depth was 50 nm. The stiffness was calculated as the proportionality coefficient in Hooke’s law: the ratio of the applied force (in N) to the indentation depth (in m). Typically, this ratio was 10^−3^ N/m, so the stiffness was expressed in pN/nm.

### 2.3. Determination of the Relative Contents of Cholesterol and Neutral Lipids

After exposure, the oocytes were fixed in a 4% buffered paraformaldehyde solution, dried and washed in phosphate–saline buffer. To determine the cholesterol content, they were stained with the fluorescent dye Fillipin III (sc-205323A, Santa Cruz Biotechnology^®^, Dallas, TX, USA); to determine the content of neutral lipids, they were stained with the fluorescent dye BODIPY 493/503 (D3922, Invitrogen^®^, Carlsbad, CA, USA) for 1.5 h at room temperature. The stained oocytes were mounted in VECTASHIELD^®^ Antifade Mounting Medium (H-1000-10, Vector Laboratories^®^, Burlingame, CA, USA) and imaged using an IX73 inverted fluorescence microscope (Olympus Corporation^®^, Tokyo, Japan) at 20× magnification. The focus during image acquisition was chosen so that the area of the oocyte was the maximum. Subsequent image analysis was carried out using the software package Fiji (https://imagej.net/Fiji, access date 14 July 2023). The area of oocytes and fluorescence intensity were determined and were compared in the experimental and corresponding control groups stained at the same time. At least three biological replicates of each group were made, and at least 100 oocytes were used for data acquisition.

### 2.4. Statistical Analysis

The results were analyzed using ANOVA using a post hoc Student’s t-test with the Bonferroni correction for multiple comparisons with a significance level of *p* < 0.05. The data are presented as the M ± SE (M—arithmetic mean; SE—standard error of the mean). Results from at least three biological replicates were used to obtain each average value.

## 3. Results

### 3.1. Oocytes’ Stiffness

**Figure 1:**
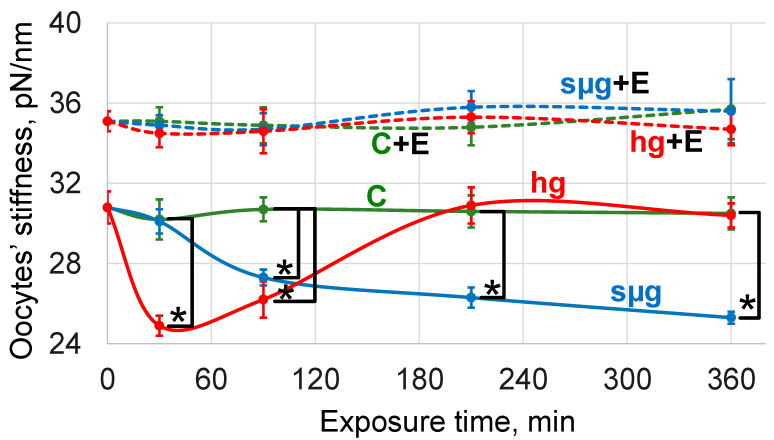
Dynamics of the oocytes’ stiffness over time of exposure under simulated weightlessness and hypergravity. Solid lines indicate the dynamics of changes in the stiffness of oocytes of flies that were cultured on a standard nutrient medium; dotted lines indicate culture on a medium with the addition of essential phospholipids. Green color—control (C), blue color—simulated microgravity (sµg), and red color—hypergravity at 2 g level (hg). “+E” marks groups of oocytes from flies cultured on a medium with 500 mg/kg essential phospholipids. *—*p* < 0.05 in comparison to the appropriate control.

The stiffness of oocytes of the control groups in the dynamics of exposure (after 30, 90, 210, and 360 min) did not differ from the initial value of the C0 group (Figure 1).

Also, as previously shown in [33], under simulated weightlessness, the stiffness of oocytes of flies that received a standard nutrient medium began to decrease after 90 min of exposure (by 12%, *p* < 0.05) and continued to fall up to 360 min, where it was below the level of the corresponding control by 18% (*p* < 0.05). Under hypergravity, the stiffness decreased after 30 min by 20% (*p* < 0.05) and remained below the corresponding control after 90 min of exposure, but after 210 min, it recovered to the initial value and did not change up to 360 min (Figure 1).

The stiffness of oocytes from flies that received essential phospholipids with a nutrient medium at the beginning of exposure (group C + E0) was higher than in the control (group C0) by 14% (*p* < 0.05). The exposure of these oocytes for up to 360 min to simulated weightlessness and hypergravity did not change cell stiffness (Figure 1).

### 3.2. Maximum Cross-Sectional Area of Oocytes

**Figure 2:**
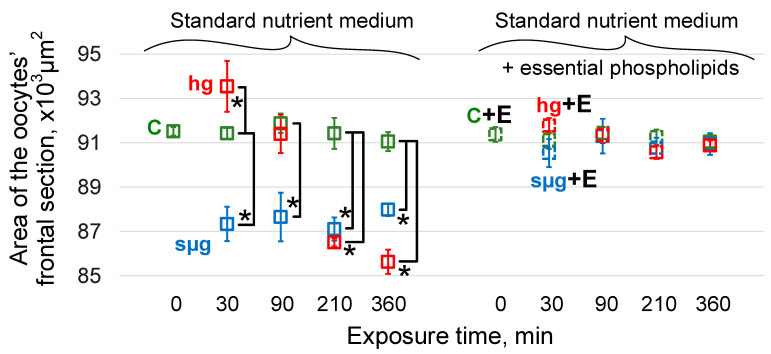
Dynamics of the area of the oocytes’ maximal cross-section over time of exposure under simulated micro- and hypergravity. As above, green color—control (C), blue color—simulated microgravity (sµg), and red color—hypergravity at 2 g level (hg). Solid lines and dotted lines (and groups with “+E”): medium without and with 500 mg/kg essential phospholipids, accordingly. *—*p* < 0.05 in comparison to the appropriate control.

The area of oocytes of the control groups in the dynamics of exposure (after 30, 90, 210, and 360 min) did not differ from the initial group C0 (Figure 2).

As previously shown [33], under simulated weightlessness after 30 min of exposure, the maximum cross-sectional area of oocytes of flies that received a standard nutrient medium decreased significantly (*p* < 0.05) and remained at this level throughout the entire exposure period (up to 360 min). Under hypergravity conditions, the oocyte area significantly increased after 30 min (*p* < 0.05) and then began to decrease; after 90 min, there were no differences from the control, but after 210 min, the area was significantly lower than the control (*p* < 0.05) and decreased even more after 360 min of exposure (Figure 2).

The area of oocytes of flies receiving essential phospholipids did not differ from the area of oocytes of flies receiving the standard nutrient medium (group C + E0 vs. group C0) up to 360 min of exposure. Moreover, under simulated weightlessness and hypergravity, there were no changes in the oocyte area in the sµg + E and hg + E groups (Figure 2).

### 3.3. Relative Cholesterol Content

**Figure 3:**
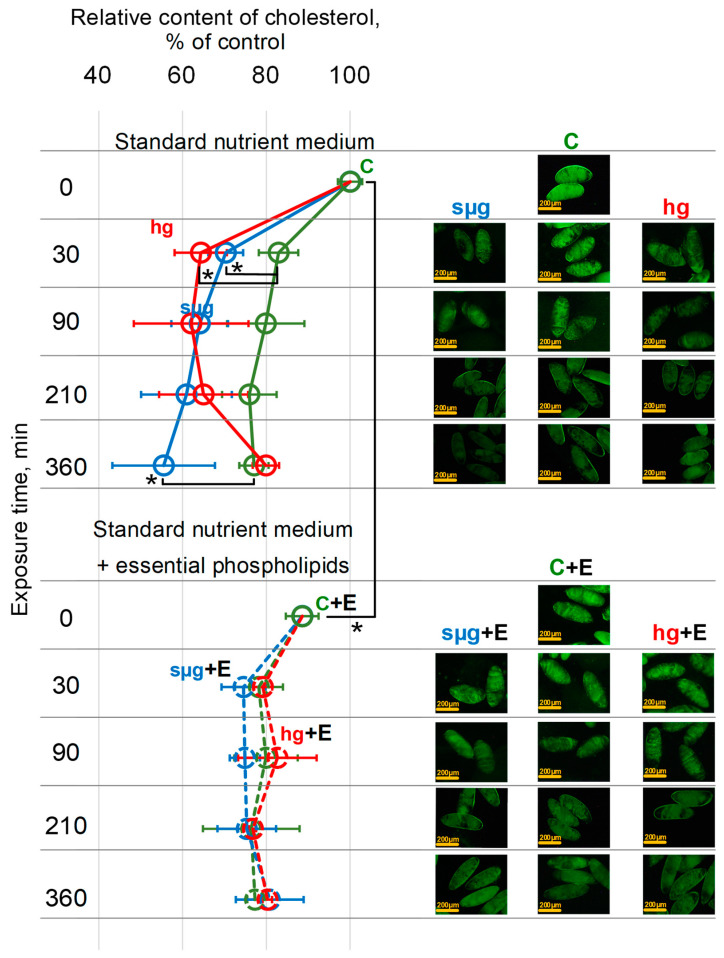
Dynamics of the relative content of cholesterol in the oocytes during exposure to simulated weightlessness and hypergravity. As above, solid lines indicate the dynamics of changes in the cholesterol content of oocytes of flies that were cultured on a standard nutrient medium: dotted lines indicate culture on a medium with the addition of essential phospholipids. Green color—control (C), blue color—simulated microgravity (sµg), and red color—hypergravity at 2 g level (hg). “+E” marks groups of oocytes from flies cultured on a medium with 500 mg/kg essential phospholipids. *—*p* < 0.05 in comparison to the appropriate control. Typical fluorescence images of oocytes stained with Fillipin III (see Materials and Methods section) are on the right panels with a 200 µm bar.

The cholesterol content in the control oocytes of flies that received a standard nutrient medium decreases with the dynamics of exposure: in the C30 group, it was lower than in the control (C0 group) by 17% (*p* < 0.05), after 90 min (C90 group) by 20 % (*p* < 0.05), after 210 min (C210 group) by 24% (*p* < 0.05), and after 360 min (C360 group) by 23 % (*p* < 0.05) (Figure 3).

The exposure of oocytes from flies that received a standard nutrient medium under simulated weightlessness led to a sharp decrease in the cholesterol content after 30 min: in the sµg30 group, it was lower than in the C30 group by 13% (*p* <0.05). Furthermore, during exposure to simulated microgravity, the relative cholesterol content in oocytes decreased less intensively and, therefore, against the background of the decrease in the control groups, there were no significant differences after 90 and 210 min. However, after 360 min (in the sµg360 group), the relative cholesterol content was lower by 22% (*p* < 0.05) compared to the corresponding control group (C360).

Similar to the sµg groups, after 30 min in hypergravity, there was a decrease in the cholesterol content relative to the corresponding C30 control by 18% (*p* < 0.05), and there were no significant differences after 90 and 210 min. At the same time, in contrast to simulated microgravity, after 360 min under hypergravity conditions, the relative content was at the level of the corresponding C360 control but increased compared to 30 min of exposure (Figure 3).

The cholesterol content in the oocytes of flies that received essential phospholipids in the C + E0 group was lower than in the C0 group (oocytes from flies that received a standard nutrient medium) by 13% (*p* < 0.05). Furthermore, after 30 min, it decreased by 10% (*p* < 0.05) (group C + E30 vs. group C + E0), and furthermore, it remained at the same level after 90, 210, and 360 min. The exposure to simulated weightlessness and hypergravity of oocytes from flies that received essential phospholipids did not change the dynamics of cholesterol reduction compared to the corresponding control group (C + E at all time points) (Figure 3).

It should be noted that in all groups of the study, when the cholesterol content decreased to a level of about 75% of the initial level (group C0), which is typical for these cells, a further decrease did not occur (Figure 3).

### 3.4. Relative Content of Neutral Lipids

**Figure 4:**
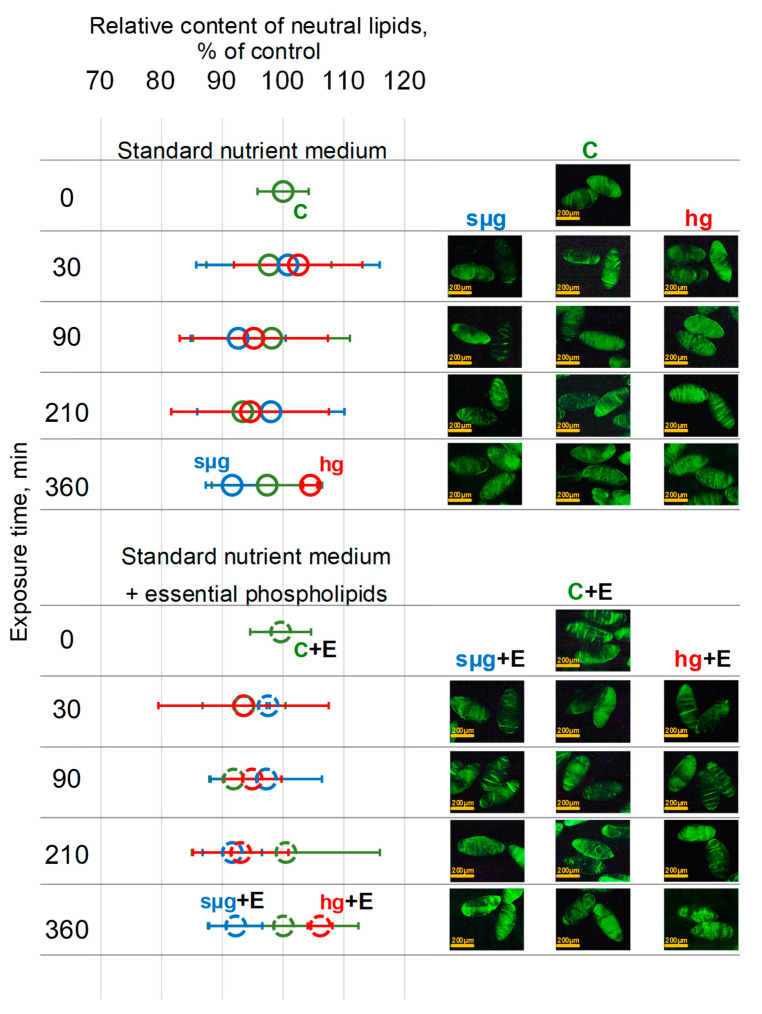
Dynamics of the relative content of neutral lipids in the oocytes during exposure to simulated weightlessness and hypergravity. As above, solid lines indicate the dynamics of changes in the oocytes of flies that were cultured on a standard nutrient medium; dotted lines indicate culture on a medium with the addition of essential phospholipids. Green color—control (C), blue color—simulated microgravity (sµg), and red color—hypergravity at 2 g level (hg). “+E” marks groups of oocytes from flies cultured on a medium with 500 mg/kg essential phospholipids. Typical fluorescence images of oocytes stained with BODIPY 493/503 (see Materials and Methods section) are on the right panels with a 200 µm bar.

Neutral lipid staining of fly oocytes was performed to assess the effects of essential phospholipids, simulated weightlessness or hypergravity, and their combined effects on the lipid profile. Neutral lipids, for example cholesteryl ester and triacylglycerol, are hydrophobic molecules that do not contain charged groups. The results obtained indicate that none of the effects led to a change in the content of neutral lipids: in all study groups, the fluorescence of stained oocytes did not change (Figure 4).

## 4. Discussion

The influence of a gravity change on the single cell still remains one of the rarely studied problems of modern cellular biophysics: it is completely unclear how a cell perceives changes in the gravity field in which it is located and how exactly different reactions to weightlessness and hypergravity are realized.

In previous works, we assumed that the cortical cytoskeleton could act as a mechanosensor, the deformation of which leads to the launch of intracellular signaling pathways [31,32]. Under weightlessness or hypergravity, various deformations (compression or stretching) occur, which leads to the dissociation of various proteins from the cortical cytoskeleton and the launch of different signaling pathways [30,33]. Therefore, considering this mechanism from a physical point of view, it can be expected that an increase in cell stiffness will lead to an increase in its resistance to deformation.

Cell stiffness can be increased via the condensation of filaments in the submembrane cytoskeleton, for example, F-actin by methyl-beta-cyclodextrin [34,35] or calyculin A [33]. In addition, changes in the cholesterol content in the cell membrane lead to cytoskeletal rearrangements [39,40,41]. It should be noted that in the cancer cell line, on the contrary, cholesterol extraction led to a decrease in the F-actin content in the submembrane cytoskeleton [34], which does not exclude the role of cytoskeleton remodeling but requires further study.

In our work, we used essential phospholipids to reduce the cholesterol content in the membranes of oocytes of the fruit fly *Drosophila melanogaster*. According to the abovementioned studies [34,35,39,40,41], we expected that since cholesterol increases the stiffness of the cell membrane, its extraction leads to a compensatory increase in the submembrane cytoskeleton. So, increasing oocyte stiffness will prevent cell deformation when exposed to conditions of simulated weightlessness and hypergravity.

Our results indicate that the receipt of essential phospholipids by flies with the nutrient medium for at least three consecutive generations leads to a significant decrease in the cholesterol content in the membranes of their oocytes. Moreover, the level of this decrease is comparable to the decrease in the cholesterol content in the oocytes of mice that received essential phospholipids per os for 6 weeks [37]. The stiffness of the oocytes of flies that received essential phospholipids was significantly higher than that of the control ones and, when exposed to simulated weightlessness and hypergravity, did not change, nor did the area, which indicates their resistance to deformation (Figure 1 and Figure 2).

We used dechorionized oocytes to avoid confusion in the interpretation of the results due to the possible role of chorion cells. Oocytes are not capable of de novo synthesis of cholesterol [42]. That is why over time the cholesterol content in oocytes’ membranes decreases due to migration along the concentration gradient into the extracellular space [43], which we observed during exposure of oocytes from both control groups (C and C + E) (Figure 3).

At the same time, the exposure to simulated weightlessness and hypergravity of oocytes from flies that received a standard nutrient medium led to a more intense loss of cholesterol from cell membranes after 30 min (Figure 3). Cholesterol rafts are anchored to the cortical cytoskeleton [44], the deformation of which can lead to the loss of this connection and the intensification of cholesterol migration into the extracellular space. By being more resistant to deformation oocytes (from flies that received essential phospholipids), this effect was prevented (Figure 3).

In our opinion, the fact of restoration of the cholesterol content to the control level in the oocytes after 360 min of exposure to hypergravity conditions (hg360 group) in the absence of the possibility of cholesterol synthesis deserves attention. Deformations of the cytoskeleton in micro- and hypergravity conditions are different: compression and stretching (Figure 2). It can be assumed that during stretching, the connection of the cytoskeleton with cholesterol rafts is broken, and cholesterol migrates not only into the extracellular space (along the concentration gradient as in the corresponding control) but also into the cell. The restoration of stiffness after 210 min (in the hg210 group) (Figure 1), even in the absence of the use of essential phospholipids, according to our previous data on the content of cytoskeletal proteins in the membrane and cytoplasmic fractions [33], can be due to both cytoskeleton remodeling and changes in expression. However, the oocyte area remains below the control level (Figure 2), which may indicate the restoration of the structure, but to a lesser volume. It should be mentioned that a change in the area can be a marker of deformation only at the first stage of exposure, and then the area reflects the formation of an adaptation pattern. The restoration of the cortical cytoskeleton structure allows the cholesterol that initially migrated into the cell to be reintegrated into the cell membrane. Therefore, we see the relative cholesterol content after 360 min of hypergravity exposure at the same level as the corresponding time control group C360 (Figure 3).

Changes in the cholesterol content, especially through the saturation of essential phospholipids in the cell membrane, can lead to changes in the lipid profile and membrane charge, which is often a negative prognostic sign [45,46,47,48]. Therefore, we assessed the content of neutral lipids both in the oocytes of the control group during the dynamics of exposure under simulated weightlessness and hypergravity and in the oocytes of flies that received essential phospholipids. We did not find any significant changes in the content of neutral lipids, which allows us to propose the use of essential phospholipids as a protective agent during exposure to an altered gravitational field (Figure 4).

Thus, the use of essential phospholipids per os in the fruit fly *Drosophila melanogaster* makes it possible to prevent the deformation of its oocytes and a sharp decrease in cholesterol in their membranes upon exposure to simulated micro- and hypergravity, without leading to negative changes in the lipid profile of the membrane.

## 5. Conclusions

So, when the force of gravity acting on a cell changes, deformations of its cytoskeleton, primarily the cortical one, occur, which leads to a change in its shape. The deformation of the cytoskeleton leads to a change in its structure and, as a result, the intensification of cholesterol loss, migration of various binding proteins, and, finally, the initiation of mechanotransduction pathways. Increasing the stiffness of the submembrane cytoskeleton helps prevent these changes. For this purpose, phospholipids with polyunsaturated fatty acids in the tail groups can be used. With an initially reduced cholesterol content in *Drosophila melanogaster* oocytes that received essential phospholipids with the nutrient medium, the cell stiffness increased, and that is why cells were more resistant to the change in gravity. That is why there were no deformations: the cross-sectional area did not change under simulated weightlessness and hypergravity. The dynamics of cholesterol reduction in this group of oocytes also did not differ from the control. Despite the initial change in the cholesterol content, the lipid profile was not changed, which allows us to consider phospholipids with polyunsaturated fatty acids in the tail groups as one of the agents for protection against the negative effects of weightlessness and overload during deep space exploration.

## Data Availability

All data generated or analyzed during this study are included in this article.
